# Supplementary material for: Toward New Epidemiological Landscapes of Trypanosoma cruzi (Kinetoplastida, Trypanosomatidae) Transmission under Future Human-Modified Land Cover and Climatic Change in Mexico
Source: Trop Med Infect Dis. 2022 Sep 2;7(9):221. doi: 10.3390/tropicalmed7090221 (PMC9503189; doi:10.3390/tropicalmed7090221)
Supplement: Supplementary file 1 [file tropicalmed-07-00221-s001.zip › tropicalmed-1836522-supplementary.pdf]

Tropical Medicine and Infectious Disease

MDPI

Table S1. Species lists of vectors and hosts species confirmed positive for *T. cruzi*.

Vectors/hosts	Especies
Vector	*Dipetalogaster maxima*
Vector	*Triatoma dimidiata*
Vector	*Triatoma longipennis*
Vector	*Triatoma mazzottii*
Vector	*Triatoma pallidipennis*
Vector	*Triatoma phyllosomus*
Vector	*Triatoma picturatus*
Vector	*Triatoma barberi*
Vector	*Triatoma brailovskyi*
Vector	*Triatoma gerstaeckeri*
Vector	*Triatoma hegneri*
Vector	*Triatoma lecticularia*
Vector	*Triatoma mexicana*
Vector	*Triatoma neotomae*
Vector	*Triatoma nitida*
Vector	*Triatoma protracta*
Vector	*Triatoma recurva*
Vector	*Triatoma rubida*
Vector	*Triatoma sp.*
Vector	*Panstrongylus rufotuberculatus*
Vector	*Rhodnius prolixus*
Sylvatic host	*Alouatta palliata*
Sylvatic host	*Alouatta villosa*
Sylvatic host	*Balantiopteryx plicata*
Sylvatic host	*Dermanura sp.*
Sylvatic host	*Heteromys desmarestianus*
Sylvatic host	*Leptonycteris curasoae*
Sylvatic host	*Macrotus waterhousii*
Sylvatic host	*Marmosa mexicana*
Sylvatic host	*Mormoops megalophylla*
Sylvatic host	*Oryzomys couesi*
Sylvatic host	*Ototylomys phyllotis*
Sylvatic host	*Peromyscus leucopus*
Sylvatic host	*Peromyscus mexicanus*
Sylvatic host	*Sigmodon toltecus*
Sylvatic-domestic host	*Artibeus jamaicensis*
Sylvatic-domestic host	*Artibeus lituratus*
Sylvatic-domestic host	*Baiomys musculus*
Sylvatic-domestic host	*Carollia sowelli*
Sylvatic-domestic host	*Choeronycteris mexicana*
Sylvatic-domestic host	*Dasypus novemcinctus*
Sylvatic-domestic host	*Dermanura phaeotis*
Sylvatic-domestic host	*Desmodus rotundus*
Sylvatic-domestic host	*Didelphis marsupialis*

Sylvatic-domestic host	*Didelphis virginiana*
Sylvatic-domestic host	*Glossophaga soricina*
Sylvatic-domestic host	*Heteromys gaumeri*
Sylvatic-domestic host	*Heteromys irroratus*
Sylvatic-domestic host	*Leptonycteris yerbabuenae*
Sylvatic-domestic host	*Mephitis mephitis*
Sylvatic-domestic host	*Micronycteris megalotis*
Sylvatic-domestic host	*Molossus rufus*
Sylvatic-domestic host	*Myotis keaysi*
Sylvatic-domestic host	*Nasua narica*
Sylvatic-domestic host	*Neotoma mexicana*
Sylvatic-domestic host	*Neotoma micropus*
Sylvatic-domestic host	*Noctilio leporinus*
Sylvatic-domestic host	*Otospermophilus variegatus*
Sylvatic-domestic host	*Peromyscus levipes*
Sylvatic-domestic host	*Peromyscus melanophrys*
Sylvatic-domestic host	*Peromyscus yucatanicus*
Sylvatic-domestic host	*Philander opossum*
Sylvatic-domestic host	*Procyon lotor*
Sylvatic-domestic host	*Pteronotus parnellii*
Sylvatic-domestic host	*Pteronotus davyi*
Sylvatic-domestic host	*Reithrodontomys fulvescens*
Sylvatic-domestic host	*Reithrodontomys gracilis*
Sylvatic-domestic host	*Rhogeessa parvula*
Sylvatic-domestic host	*Sigmodon hispidus*
Sylvatic-domestic host	*Sturnira lilium*
Sylvatic-domestic host	*Sturnira ludovici*
Sylvatic-domestic host	*Sturnira parvidens*
Sylvatic-domestic host	*Uroderma bilobatum*
Domestic host	*Canis familiaris*
Domestic host	*Felis catus*
Domestic host	*Bos taurus*
Domestic host	*Capra hircus*
Domestic host	*Equus asinus*
Domestic host	*Equus caballus*
Domestic host	*Ovis aries*
Domestic host	*Oryctolagus cuniculus*
Domestic host	*Sus domesticus*
Synanthropic host	*Rattus norvegicus*
Synanthropic host	*Rattus rattus*
Synanthropic host	*Mus musculus*

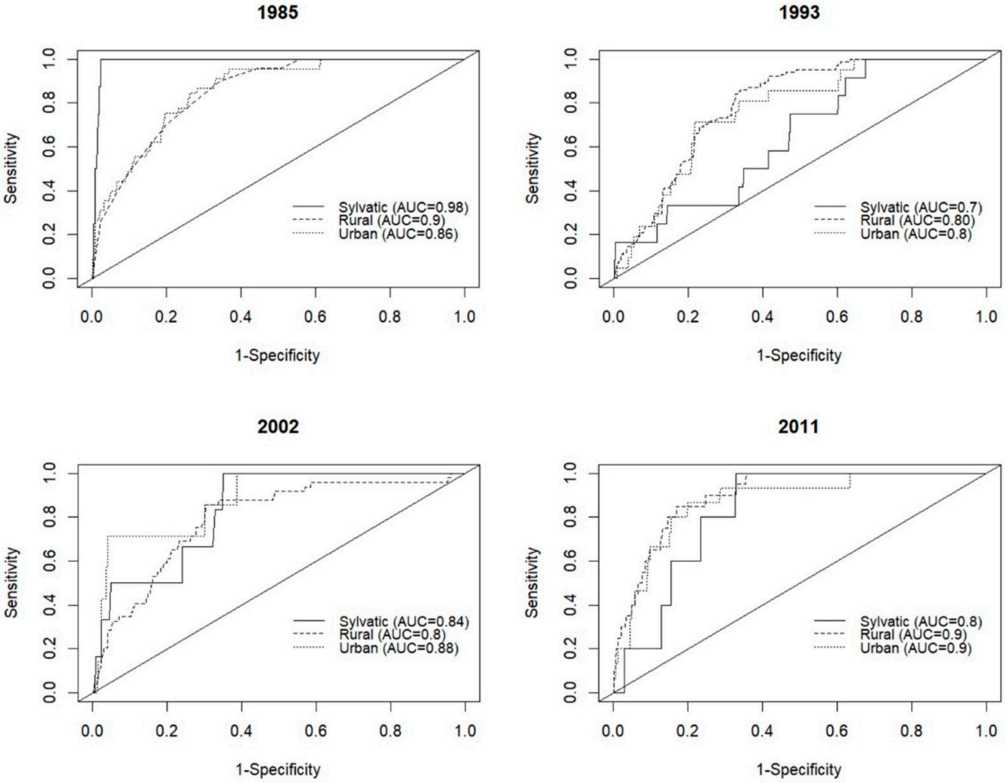

Figure S1. AUC values of 1985 baseline LUCC-Climate model and projections to 1993, 2002, and 2011 for each *T. cruzi* transmission cycle.
